# Supplementary material for: Comparative genomics provides new insights into the diversity, physiology, and sexuality of the only industrially exploited tremellomycete: Phaffia rhodozyma
Source: BMC Genomics. 2016 Nov 9;17:901. doi: 10.1186/s12864-016-3244-7 (PMC5103461; doi:10.1186/s12864-016-3244-7)
Supplement: Additional file 6: — List of orphan genes with links to PFAM (related to Additional file 1: Table S1). (ZIP 1428 kb) [file 12864_2016_3244_MOESM6_ESM.zip › BLAST_HTML_FTR/G01559_P.html]

BLAST Search Results


```
BLASTP 2.2.27+


Reference:
Stephen F. Altschul, Thomas L. Madden, Alejandro A. Schäffer,
Jinghui Zhang, Zheng Zhang, Webb Miller, and David J. Lipman (1997),
"Gapped BLAST and PSI-BLAST: a new generation of protein database
search programs", Nucleic Acids Res. 25:3389-3402.


Reference for
composition-based statistics:
Alejandro A. Schäffer, L. Aravind, Thomas L. Madden, Sergei
Shavirin, John L. Spouge, Yuri I. Wolf, Eugene V. Koonin, and
Stephen F. Altschul (2001), "Improving the accuracy of PSI-BLAST
protein database searches with composition-based statistics and
other refinements", Nucleic Acids Res. 29:2994-3005.


Database: nr
           71,551,133 sequences; 26,053,659,533 total letters


Query= G01559_P

Length=841
                                                                      Score     E
Sequences producing significant alignments:                          (Bits)  Value

emb|CDZ98378.1|  hypothetical protein [Xanthophyllomyces dendrorh...  1652    0.0  


 >emb|CDZ98378.1| hypothetical protein [Xanthophyllomyces dendrorhous]
Length=840

 Score = 1652 bits (4279),  Expect = 0.0, Method: Compositional matrix adjust.
 Identities = 839/840 (99%), Positives = 840/840 (100%), Gaps = 0/840 (0%)

Query  1    MTSHGSVGSLFKENLSSESLPETELFILIGRNPIQEPQPPGEPRPEHSHPTQPIRTQTIR  60
            MTSHGSVGSLFKENLSSESLPETELFILIGRNPIQEPQPPGEPRPEHSHPTQPIRTQTIR
Sbjct  1    MTSHGSVGSLFKENLSSESLPETELFILIGRNPIQEPQPPGEPRPEHSHPTQPIRTQTIR  60

Query  61   KPSFRRKQPPRRKIDLPTALVLPQIALPDQVQPSSLSSPKILGGWNFLKRQNGPLSSPSV  120
            KPSFRRKQPPRRKIDLPTALVLPQIALPDQVQPSSLSSPKILGGWNFLKRQNGPLSSPSV
Sbjct  61   KPSFRRKQPPRRKIDLPTALVLPQIALPDQVQPSSLSSPKILGGWNFLKRQNGPLSSPSV  120

Query  121  PLPRESRTSLTSPKRRLSSTDESDRSRVSTPVRSIRPAQSRSSTWTDNVPTVRSLAQRSP  180
            PLPRESRTSLTSPKRRLSSTDESDRSRVSTPVRSIRPAQSRSSTWTDNVPTVRSLAQRSP
Sbjct  121  PLPRESRTSLTSPKRRLSSTDESDRSRVSTPVRSIRPAQSRSSTWTDNVPTVRSLAQRSP  180

Query  181  SAEYTRPLSTSLYPLSLSTATDSLTTVSPVSLASPKVVVPLAVVSKDNPRGGKFGLFVRR  240
            SAEYTRPLSTSLYPLSLSTATDSLTTVSPVSLASPKVVVPLAVVSKDNPRGGKFGLFVRR
Sbjct  181  SAEYTRPLSTSLYPLSLSTATDSLTTVSPVSLASPKVVVPLAVVSKDNPRGGKFGLFVRR  240

Query  241  GSVSPGQERTGFFAGFAESSTNKKERSKTEKQQKKGLGKGNESKKDLGLIKQLSNTSSSS  300
            GSVSPGQERTGFFAGFAESSTNKKERSKTEKQQKKGLGKGNESKKDLGLIKQLSNTSSSS
Sbjct  241  GSVSPGQERTGFFAGFAESSTNKKERSKTEKQQKKGLGKGNESKKDLGLIKQLSNTSSSS  300

Query  301  YSSSSVNNRKRSFFSTQPVVTNTTEHTRTPKTTIQWGPTAEEGRHTALTTASPPPPPSVF  360
            YSSSSVNNRKRSFFSTQPVVTNTTEHTRTPKTTIQWGPTAEEGRHTALTTASPPPPPSVF
Sbjct  301  YSSSSVNNRKRSFFSTQPVVTNTTEHTRTPKTTIQWGPTAEEGRHTALTTASPPPPPSVF  360

Query  361  TDAPVSSPSQLQIPLSRTISPLSNPPLSVSPLPIASLPASYSSVSIRSDSASSFSDEEFP  420
            TDAPVSSPSQLQIPLSRTISPLSNPPLSVSPLPIASLPASYSSVSIRSDSASSFSDEEFP
Sbjct  361  TDAPVSSPSQLQIPLSRTISPLSNPPLSVSPLPIASLPASYSSVSIRSDSASSFSDEEFP  420

Query  421  PRSPKSQRSQVYSIANGSASTTPTPSEQEDQHRFSAHRMPIASVFRERSSSPLRNELLGR  480
            PRSPKSQRSQVYSIANGSASTTPTPSEQEDQHRFSAHRMPIASVFRERSSSPLRNELLGR
Sbjct  421  PRSPKSQRSQVYSIANGSASTTPTPSEQEDQHRFSAHRMPIASVFRERSSSPLRNELLGR  480

Query  481  SPRSRDLNIAAETPVEAPVNIQTGRLKQAPTAPERTGSTSPSKHPWWKPGTGRNSPSLRT  540
            SPRSRDLNIAAETPVEAPVNIQTGRLKQAPTAPERTGSTSPSKHPWWKPGTGRNSPSLRT
Sbjct  481  SPRSRDLNIAAETPVEAPVNIQTGRLKQAPTAPERTGSTSPSKHPWWKPGTGRNSPSLRT  540

Query  541  DLPAPVSIRMDGQASDSVDNTVGIRRGTANGSTSGRRRSIFGSPGSFFSLGGGSNAGSVG  600
            DLPAPVSIRMDGQASDSVDNTVGIRRGTANGSTSGRRRSIFGSPGSFFSLGGGSNAGSVG
Sbjct  541  DLPAPVSIRMDGQASDSVDNTVGIRRGTANGSTSGRRRSIFGSPGSFFSLGGGSNAGSVG  600

Query  601  ESIKGRARRASDAPLTLNRRESDMFSQAAPPATSTTTAKTVTQRGFEVTSRESYRPPNAQ  660
            ESIKGRARRASDAPLTLNRRESDMFSQAAPPATSTTTAKTVTQRGFEVTSRESYRPPNAQ
Sbjct  601  ESIKGRARRASDAPLTLNRRESDMFSQAAPPATSTTTAKTVTQRGFEVTSRESYRPPNAQ  660

Query  661  PADNNGNIHVLSVVNDAASSIAIDTATCTDADTTTITDADADTLPTPPDSTVSTCPPRRL  720
            PADNNGNIHVLSVVNDAASSIA+DTATCTDADTTTITDADADTLPTPPDSTVSTCPPRRL
Sbjct  661  PADNNGNIHVLSVVNDAASSIAVDTATCTDADTTTITDADADTLPTPPDSTVSTCPPRRL  720

Query  721  PSPVARPTEVITIPAGPSRPSNLTASRFTPNGPGLTSIMIHSSKAAKTWVPLSKRAPRPP  780
            PSPVARPTEVITIPAGPSRPSNLTASRFTPNGPGLTSIMIHSSKAAKTWVPLSKRAPRPP
Sbjct  721  PSPVARPTEVITIPAGPSRPSNLTASRFTPNGPGLTSIMIHSSKAAKTWVPLSKRAPRPP  780

Query  781  LTPPLSTADQARYPLDGEPARPQQEGSEEFSPASSSGSEAGDENGAHGWIGVPELGRPVR  840
            LTPPLSTADQARYPLDGEPARPQQEGSEEFSPASSSGSEAGDENGAHGWIGVPELGRPVR
Sbjct  781  LTPPLSTADQARYPLDGEPARPQQEGSEEFSPASSSGSEAGDENGAHGWIGVPELGRPVR  840


Lambda      K        H        a         alpha
   0.309    0.125    0.361    0.792     4.96 

Gapped
Lambda      K        H        a         alpha    sigma
   0.267   0.0410    0.140     1.90     42.6     43.6 

Effective search space used: 9946330690293


  Database: nr
    Posted date:  Sep 23, 2015 12:05 AM
  Number of letters in database: 26,053,659,533
  Number of sequences in database:  71,551,133


Matrix: BLOSUM62
Gap Penalties: Existence: 11, Extension: 1
Neighboring words threshold: 11
Window for multiple hits: 40
```
